# Three Decades of Research on Recombinant Collagens: Reinventing the Wheel or Developing New Biomedical Products?

**DOI:** 10.3390/bioengineering7040155

**Published:** 2020-12-02

**Authors:** Andrzej Fertala

**Affiliations:** Department of Orthopaedic Surgery, Sidney Kimmel Medical College, Thomas Jefferson University, Curtis Building, Room 501, 1015 Walnut Street, Philadelphia, PA 19107, USA; axf116@jefferson.edu; Tel.: +1-215-503-0113

**Keywords:** recombinant collagen, gelatin, biomaterials, tissue engineering

## Abstract

Collagens provide the building blocks for diverse tissues and organs. Furthermore, these proteins act as signaling molecules that control cell behavior during organ development, growth, and repair. Their long half-life, mechanical strength, ability to assemble into fibrils and networks, biocompatibility, and abundance from readily available discarded animal tissues make collagens an attractive material in biomedicine, drug and food industries, and cosmetic products. About three decades ago, pioneering experiments led to recombinant human collagens’ expression, thereby initiating studies on the potential use of these proteins as substitutes for the animal-derived collagens. Since then, scientists have utilized various systems to produce native-like recombinant collagens and their fragments. They also tested these collagens as materials to repair tissues, deliver drugs, and serve as therapeutics. Although many tests demonstrated that recombinant collagens perform as well as their native counterparts, the recombinant collagen technology has not yet been adopted by the biomedical, pharmaceutical, or food industry. This paper highlights recent technologies to produce and utilize recombinant collagens, and it contemplates their prospects and limitations.

## 1. Introduction

Proteins, including insulin, various growth factors, enzymes, vaccines, and antibodies serve as irreplaceable therapeutics to prevent and treat diverse diseases. Moreover, proteins are used as vital research and diagnostic tools [1].

While in the past, proteins utilized in medicine and research had to be isolated from natural sources, such as animal tissues, plants, bacteria, and marine organisms, to name a few, today, their recombinant forms are also available [2,3]. The ability to produce the recombinant variants of native proteins provides therapeutics of high purity, batch-to-batch consistency, biocompatibility, low immunogenicity, and ample supply. Technologies to produce recombinant proteins are often the only sustainable source of therapeutic proteins (e.g., humanized monoclonal antibodies), and generating recombinant proteins can cost less than isolating proteins from natural sources. Thus, the design and production of recombinant proteins for biomedical applications and research are crucial biotechnology areas today [1].

Unlike therapeutic recombinant growth factors, protein hormones, enzymes, soluble receptors, and therapeutic antibodies, which are biologically active at relatively low concentrations, collagens naturally aggregate into massive insoluble structures. As a result, collagen-based products require large amounts of collagen material. However, the production of recombinant collagen variants is challenging due to the complex structure of collagens, whose intracellular biosynthesis depends on collagen-specific chaperones and modifying enzymes. The need for distinct types of collagens that form tissue-specific structures, including fibrils, networks, transmembrane structures, and others, exacerbates this challenge.

## 2. Collagens: The Cornerstones of Tissue Architecture

Like other recombinant proteins produced to substitute for their native counterparts, recombinant collagens must have crucial physicochemical characteristics that match those seen in collagens that build healthy tissues [4]. The following paragraphs highlight the crucial features of collagens needed for these proteins’ mechanical and biological functions.

### 2.1. Biosynthesis of Triple-Helical Collagen Molecules

The family of collagenous proteins includes about 28 diverse members [5]. Despite their heterogeneity, various collagen types share a few common characteristics that distinguish them from other proteins. First, unlike the globular proteins, individual collagen molecules are shaped as extended rod-like structures. Second, each collagen molecule consists of three collagen α-chains folded into the triple-helical structure; depending on the collagen type, the triple helices may comprise identical chains (homotrimers) or different chains (heterotrimers). Third, regardless of the collagen type, each collagen α-chain consists of the Gly-X-Y motif repeats. Any amino acid residue may occupy the X and the Y positions of the Gly-X-Y triplets. However, the Gly residues at every third position are mandatory to allow the folding of the α-chains into a compact triple-helical conformation. While in the fibril-forming collagens, including types I, II, and III, the Gly-X-Y triplets form uninterrupted repeats, in other collagen types, including types IV, VII, and IX, stretches of amino acid sequences without the Gly-X-Y pattern interrupt the Gly-X-Y repeats. Those intervening sequences form flexible hinges that are needed for the specific functions of the collagen types that harbor them [5].

The formation of collagen triple helices starts with the nascent collagen α-chains encoded by specific genes. Before the α-chains fold into the triple-helical conformation, they undergo posttranslational modifications. While prolyl-4-hydroxylase (P4H) hydroxylases proline residues present at the -Y- position of the Gly-X-Y triplets, lysyl hydroxylase (LH) hydroxylases many lysine residues present at the -Y- positions. Following hydroxylation, some of the hydroxylysine residues are glycosylated.

The hydroxylation of proline and lysine residues is obligatory, and aberrations of this process alter the formation of stable triple helices and fibrils. Poorly formed collagen triple helices accumulate inside cells, causing unfolded protein response, endoplasmic reticulum stress, and apoptosis [6].

**Figure 1:**
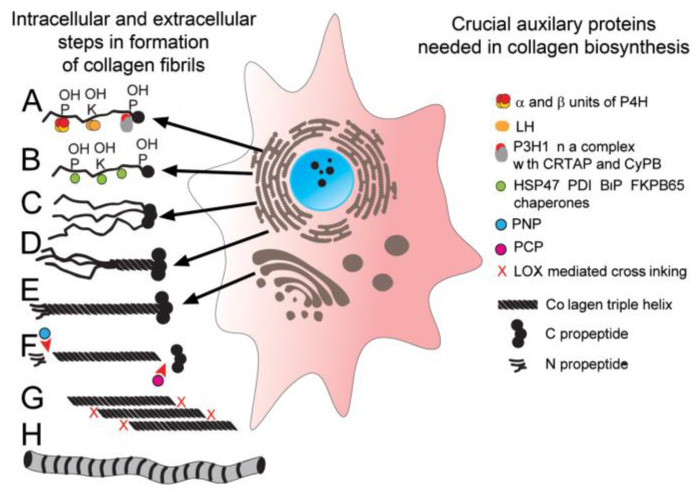
A schematic of intracellular and extracellular molecules and processes that control the formation of collagen fibrils. (**A**) Biosynthesis and post-translational modifications of individual procollagen α-chains; during this process, selected proline and lysine residues are hydroxylated. (**B**) The interaction of procollagen chains with protein chaperones that control the folding of triple helices. (**C**,**D**) The selection of procollagen chains and nucleation of triple helices. (**E**) Translocation and secretion of procollagen molecules into the extracellular space. (**F**) Cleavage of the N propeptide by PNP and C propeptides by PCP. (**G**,**H**) Assembly of collagen molecules into fibrils and formation of covalent cross-links (X). Symbols: P; proline residues, K; lysine residues, P4H; prolyl 4-hydroxylase, LH; lysyl hydroxylase, P3H1; prolyl 3-hydroxylase, CRTAP; cartilage-associated protein, CyB; cyclophilin B, HSP47; heat shock protein 47, PDI; disulfide isomerase, FKPB65; immunophilin, PNP; procollagen N proteinase, PCP; procollagen C proteinase.

Besides P4H and LH, the biosynthesis of functional collagen molecules may require the participation of other modifying enzymes, including prolyl-3-hydroxylase (P3H). Furthermore, the proper folding of stable collagens depends on protein chaperones that control the intracellular folding of collagen triple helices. A group of crucial chaperones includes heat shock protein 47 (HSP47), heat shock 70 kDa-related luminal binding protein (BiP), and the β subunit of P4H (PDI, also referred to as disulfide isomerase) [5]. Figure 1 presents crucial players in the biosynthesis of collagen molecules and their assembly into fibrils.

Following biosynthesis and posttranslational modifications, specific collagen α-chains aggregate to form the triple-helical structure. Although one cell may produce many collagen types, a complex chain selection mechanism ensures that a specific collagen type contains only a defined α-chain set. For instance, dermal fibroblasts produce collagen I, collagen III, collagen IV, collagen VII, and some additional collagen types. Among these collagens, collagen I and collagen IV are heterotrimers comprising α1(I)_2_/α2(I) chains (collagen I) and α1(IV)_2_/α2(IV) chains (collagen IV). In contrast, homotrimeric collagen III comprises α1(III)_3_ chains, and homotrimeric collagen VII comprises α1(VII)_3_ chains [5].

### 2.2. Diverse Architectures of Collagen-Rich Matrices

The biosynthesis of procollagen molecules and their secretion into the extracellular space is the starting point in the assembly of complex architectures that define connective tissue. Although various collagen types share similar triple-helical conformations, they form various supramolecular assemblies, including cylindrical fibrils and net-like structures [5]. These assemblies interact with each other to form 3D architectures able to perform unique tissue-specific functions. Examples of these architectures include fibrils organized into parallel bundles of fibers that form ligaments and tendons; orthogonal lamellae formed by uniform-diameter fibrils that build transparent corneas; collagen II-based fibrils that form articular cartilage; and thin collagen III-based filaments that form many reticular connective tissues. Additionally, the sheet-like structure of basement membranes comprises collagen IV molecules that interact via globular ends. Some basement membranes also comprise anchoring fibrils containing collagen VII molecules that maintain the structural integrity of many tissues and organs, including skin, esophagus, and elements of the eye [5].

The formation of initial patterns of those architectures occurs during embryonic development. Although mechanisms that drive the spatial organization of the collagenous structures may involve cells and mechanical cues, the amino acid sequences of the collagen chains themselves store the essential information about the assembly of collagen molecules into fibrils and networks [7,8,9,10].

### 2.3. Self-Assembly of Collagens: Paradigms of Collagen I Fibrils, Collagen VII Anchoring Fibrils, and Collagen IV Networks

Procollagens in which the globular propeptides flank the triple-helical domain are precursors of collagen molecules. For instance, the N-terminal and the C-terminal propeptides flank the triple helical regions of the fibrillar collagen types I, II, and III. Similarly, the non-collagenous (NC) NC1 and NC2 propeptides flank the collagen VII triple helix [5]. Studies demonstrated that these globular propeptides have numerous functions, including collagen chain selection, folding of triple helices, and secretion of procollagen molecules. Besides, propeptides significantly increase collagen molecules’ solubility, thereby preventing their premature aggregation into insoluble complexes [11].

Following secretion of the fibril-forming procollagens into the extracellular space, procollagen N proteinase and procollagen C proteinase cleave the propeptides. The cleavage exposes the very ends of collagen molecules, called telopeptides, that mediate the collagen–collagen binding interactions. These site-specific interactions aggregate the collagen molecules into the fibrils that build connective tissues [12,13]. A precise, D-periodic alignment of individual collagen molecules that build the fibrils enables covalent cross-linking between some lysine and hydroxylysine residues in adjoining collagen molecules. Lysyl oxidases (LOX) catalyze the formation of these cross-links. The cross-links strengthen the fibrils, allowing connective tissues to function correctly [14].

The basement membrane networks’ assembly depends on binding interactions between the NC1 domains of collagen IV [10,15]. Similarly, the formation of anchoring fibrils within the dermal basement membrane depends on the dimerization of the NC2 domains of collagen VII molecules. Here, procollagen C proteinase first cleaves these domains, which then dimerize in the anti-parallel fashion. Subsequently, the formation of intermolecular disulfide bonds stabilizes the collagen VII–collagen VII dimers that bundle together to form the anchoring fibrils [16].

Many mutations have been observed to destabilize the collagen triple helices, prevent their self-assembly, or weaken collagen-rich architectures’ structure, indicating that proper amino acid sequences are crucial for collagens to function correctly. For instance, mutations in collagen I cause osteogenesis imperfecta, a brittle bone disease, which alters bone mineralization and limits skeletal growth. Similarly, mutations in collagen IV weaken the basement membranes of kidneys, causing Alport syndrome. Mutations in collagen VII cause epidermolysis bullosa, a blistering disease whose pathomechanisms involve aberrations of the anchoring fibrils [17].

The above examples indicate that when collagen sequences change, the collagen may lose its vital functions. Consequently, genetic approaches to engineering recombinant collagen variants must preserve crucial features of these proteins to allow them to function when used in biomedical applications.

### 2.4. Collagens as Signaling Molecules

**Figure 2:**
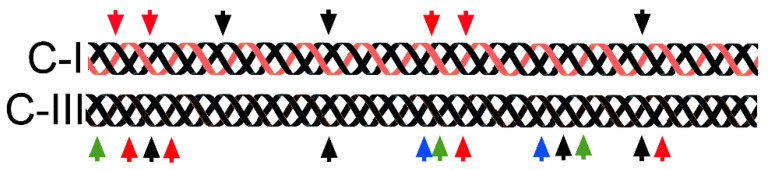
Sites of binding of collagen-specific receptors in collagen I (C-I) and collagen III (C-III). Red arrows; integrin-binding sites, black arrows; discoidin domain receptor (DDR)-binding sites, green arrows; glycoprotein VI (GPVI)-binding sites, blue arrows; leukocyte-associated immunoglobulin-like receptor 1 (LAIR1)-binding sites.

In addition to the structural functions, collagens play crucial roles as signaling molecules. Studies on mechanisms of binding interactions between collagen molecules and cells demonstrated that triple-helical regions include distinct domains, usually formed by only a few amino acid residues that interact specifically with collagen-specific cell receptors. These receptors include integrins, discoidin domain receptors (DDRs), glycoprotein VI (GPVI), or leukocyte-associated immunoglobulin-like receptor 1 (LAIR-1) (Figure 2). They not only recognize defined collagen sites, but some of them (e.g., GPVI) require incorporation of collagen I and collagen III molecules into fibrils. Although some of the collagen-specific receptors may also bind to the linear (i.e., not triple-helical) peptides corresponding to the native binding sites, the binding interactions are usually weak and may not trigger tissue-specific cell responses.

**Figure 3:**
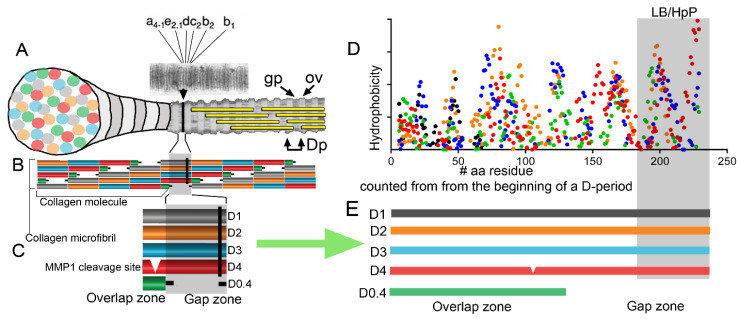
A schematic explaining the formation of the fibril-specific features formed due to aggregation of individual collagen molecules. The schematic illustrates the formation of the hydrophobic cluster (indicated by the black line (**|**) across the fibril in (**A**–**C**)). (**A**) A banding pattern of a positively stained collagen fibril. Defined bands (a, b, c, d, and e) are indicated. Please note that the hydrophobic cluster is located between c2 and d bands. Gaps (gp), overlaps (ov), and D-period (Dp) regions are visible. (**B**,**C**) Detailed representation of the gap and overlap zones with the hydrophobic cluster indicated as a black line (**|**). Additionally, MMP1 cleavage site present in the D4 period is indicated. (**D**) A hydrophobicity plots of overlapping D-periods (**C**,**E**) [19]. The highlighted zone indicates the unique lipid-binding (LB) region studied here and characterized by poor content of hydroxyproline residues (HpP). This zone corresponds to the region indicated by the black line (|) in (**A**–**C**).

As indicated earlier, the self-assembly of collagen molecules arranged in a D-staggered fashion drives the formation of fibrils. Orderly aggregation creates novel physicochemical qualities of the fibrils’ surface that arise from the clustering of the amino acid residues belonging to the interacting collagen molecules. These novel qualities, including topography, charge, or hydrophobic properties, create fibril-specific markers recognized by cellular receptors (Figure 3). Moreover, these fibril-specific markers provide unique binding sites for various macromolecules, including proteoglycans, glycosaminoglycans, growth factors, and others, thereby arranging them in the tissue-specific fashion [18].

## 3. Practical Utilization of Collagens

### 3.1. Applications of Collagens

Because of their roles as biological scaffolds, collagens are attractive materials for fabricating products for tissue engineering, wound healing, and drug delivery [20,21]. Furthermore, vaccines and other pharmaceutical products utilize gelatins, which are denatured forms of collagens, as stabilizers [22,23]. The polymeric nature of gelatin and its ability to bind various therapeutic compounds renders this collagen product an attractive carrier in many pharmaceutical applications. Moreover, gelatin is also applied as a gelling agent in food products [24,25,26,27,28].

At present, animal tissues, notably bovine hides, are the primary source of collagen material for most applications. Other sources include porcine and fish tissues. The main form of collagen used for these applications is collagen I [29,30,31,32].

### 3.2. Potential Limitations of Collagens Isolated from the Natural Sources

Despite the wide use of products manufactured from natural collagens, concerns exist about their side effects [33,34,35]. In the past, a possibility of transmitting bovine spongiform encephalopathy, also known as mad cow disease, was a particularly worrisome concern [36]. However, eliminating bovine neurological tissues as a potential source of collagen material, and utilizing bovine spongiform encephalopathy-free cattle as the sole source of collagen material, reduced these concerns significantly. Furthermore, future utilization of genetically engineered cattle lacking prion protein would offer a safe source of collagen-based materials [37].

Other concerns about using the animal-derived collagen products are associated with their potential antigenicity, defined as the ability to interact with secreted antibodies, and immunogenicity, defined as the ability to induce the immune response. While the amino acid sequences of the triple-helical regions of collagen molecules from animals and humans share significant similarities, substantial differences characterize their telopeptide regions. Consequently, telopeptides are responsible for the majority of collagen antigenicity. Meanwhile, researchers suggested that cryptic epitopes, generated by enzymatic degradation or denaturation of triple helices of the collagen-based materials, may also interact with antibodies [38].

Scientists believe that primary exposure to exogenous collagen is dietary and about 2% to 4% of the total population has an inherent immunity, i.e., allergy, to bovine collagen I [39,40,41,42]. When compared with the 10% to 15% of the population sensitive to nickel or 6% to 17% of the population sensitive to latex, the sensitivity rate to bovine collagen I is relatively low [43,44,45].

Because of the large body of data for the use of collagen-based products, most notably the injectable collagen material used for soft tissue augmentation, we know that 2% to 4% of patients show hypersensitivity to the injected material. This percentage is consistent with the overall preexisting sensitivity to collagen (see above) observed in the general population. In fewer than 3% of cases, patients develop adverse reactions to foreign collagen materials, including granuloma and localized inflammation. These reactions, however, usually subside within a few months and never last longer than a year [38].

Although researchers have pointed to collagen telopeptides as the most probable regions causing immune responses in human patients, the evidence is not clear. As discussed by Lynn et al., no documented differences exist between human responses to products manufactured from collagens that include telopeptides and those without them [38].

Evidence so far suggests that biomedical products manufactured using animal-derived collagens do not present any significant danger to the human recipients [46]. However, some concerns still exist about the potential negative impacts of non-collagenous molecules that co-purify with animal-derived collagen. Furthermore, some controversies exist about a potential induction of autoimmunity in humans by products containing collagen II [47]. Although most collagen-based products mainly include collagen I and collagen III, future applications, e.g., cartilage engineering, may require collagen II-based materials. This possibility will require further studies to clarify the potential for collagen II-associated autoimmunity.

## 4. Recombinant Collagens

The application of recombinant human collagens in research and medicine, as well as drug, food, and cosmetic industries, offers an attractive alternative to the use of the animal-derived collagen materials. Despite the overall safety of the animal collagens, the human collagens offer ultimate biocompatibility and safety. Moreover, technologies to produce recombinant collagens would potentially provide quantities of less abundant collagen types that would be impossible to isolate from tissues [24,48]. Furthermore, technologies to produce recombinant collagens may be utilized to produce unique collagenous proteins that correspond to those from other animal groups, including avian and marine species.

To address the need for human collagens, about three decades ago, researchers initiated studies on producing these proteins using recombinant DNA technology. Initially, mammalian cells were used to express full-length collagens or their fragments [49,50,51,52,53]. Subsequently, other expression systems were employed to produce collagen types, collagen fragments, and modified collagen variants [54].

### 4.1. Prerequisites for Engineering Mammalian Recombinant Collagens

As noted, a few crucial characteristics define native mammalian collagens’ ability to serve as structural and cell signaling molecules. These characteristics include thermostable triple-helical conformation with the correct composition of type-specific α-chains, proper posttranslational modifications of the chains, correct processing of propeptides, and the ability to form the supramolecular assemblies. Because these characteristics largely depend on the presence of a set of collagen-modifying enzymes, it is critical to select expression systems in which these enzymes are active.

In contrast, collagen-like proteins found in some bacteria and cocoons of a group of hymenopteran insects do not require hydroxyproline residues to maintain stability. This characteristic makes them potentially attractive substitutes for mammalian collagens in some biomedical applications [55,56].

### 4.2. Collagen Expression Systems

The first systems for producing recombinant collagens utilized mammalian cells that expressed native collagen-modifying enzymes. Procollagens produced in these cells, including procollagen I and procollagen II, had normal thermostability, and their proline and lysine residues were correctly hydroxylated. Moreover, these proteins were glycosylated and processed by procollagen N proteinase and C proteinase [53]. With proper modifications and enzymatic processing, these native-like recombinant collagens assembled into well-organized fibrils [57].

The relatively low yield and potentially high cost of the collagen-based products is a crucial limitation of mammalian cell-based expression systems in collagen production for biomedical, cosmetic, and pharmaceutical applications. To circumvent this problem, scientists tested systems compatible with industrial-scale production. These systems relied on yeast, insect cells, and bacteria. Furthermore, researchers investigated plants, including tobacco, barley, and corn, as potential collagen-producing factories. Experiments with animals demonstrated the feasibility of producing collagens in the mammary glands of transgenic mice and eggs of transgenic chickens [58,59,60,61,62,63,64,65].

Scientists also used recombinant and chemical methods to synthesize short collagen-derived peptides for research and tissue repair applications. In one example, a synthetic 15-mer peptide derived from the α1(I) chain serves as an ingredient in a material for bone repair [66,67]. Although chemical synthesis is an attractive method to produce collagen fragments, these fragments lack crucial collagen characteristics, including triple-helical conformation, modifications of proline and lysine residues, and resistance to enzymatic degradation. Employing a biological system for the production of recombinant collagen-derived peptides may provide an attractive analog for their synthetic counterparts [68,69].

Initially, a common challenge for the large-scale production systems was the lack of native enzymes needed to produce stable collagen molecules. To circumvent this problem, scientists co-expressed the collagen-coding genes with those that encode subunits of P4H. Overall, this approach was successful, and the recombinant collagens co-expressed with P4H are triple-helical and thermostable [70,71]. It is likely that other crucial enzymes, including prolyl-3-hydroxylase, will also have to be considered to ensure full functionality of recombinant collagens. Moreover, future expression systems should take into account the need for glycosylation of recombinant collagens. As hydroxylysine residues are the main sites of collagen glycosylation, co-expression of lysyl hydroxylases may help in the production of recombinant collagen variants with properties closely resembling those of their native counterparts.

Although the literature describes many expression systems, those employed today for the large-scale production of collagen I rely on yeasts and plants [58,71,72,73]. For instance, FibroGen, Inc. (San Francisco, CA, USA) manufactures recombinant collagens in yeast cells, while CollPlant Ltd. (Rehovot, Israel) employs tobacco plants as collagen-producing factories. According to published literature, these companies mainly focus on collagen I and collagen III for manufacturing biomedical products, including artificial corneas, injectable constructs, and wound-dressing materials [74,75,76]. They use proteolytic enzymes to extract collagens from the hosts’ crude materials [77]. While the enzymatic digestion removes the bulk of contaminating proteins, it also destroys procollagen propeptides and telopeptides. Because of the lack of intact telopeptides, the recombinant collagens isolated with current technologies are not able to form proper fibrillar assemblies similar to those seen in native tissues [12,13,78].

While the lack of telopeptides should not present significant problems for using enzymatically processed collagens to produce gelatin, gels containing non-denatured collagen molecules, and unorganized fibrillar matrices, the presence of telopeptides is critical for the formation of native-like structures intended to mimic the biological and mechanical functions of collagens. This notion is particularly important considering that in native fibrils, the C-terminal telopeptides and nearby domains form significant matrix–matrix and cell–matrix interaction regions [18,79]. For instance, recent research indicates that the tyrosine cluster present in the C-terminal telopeptides plays a significant role in the interaction of collagen fibrils with the human osteoclast-associated receptor (OSCAR) that plays an important role in the modulation of matrix remodeling and in antigen recognition [80,81,82].

Table 1 summarizes crucial expression systems for the production of recombinant collagen variants.

### 4.3. Recombinant Collagen Variants

**Figure 4:**
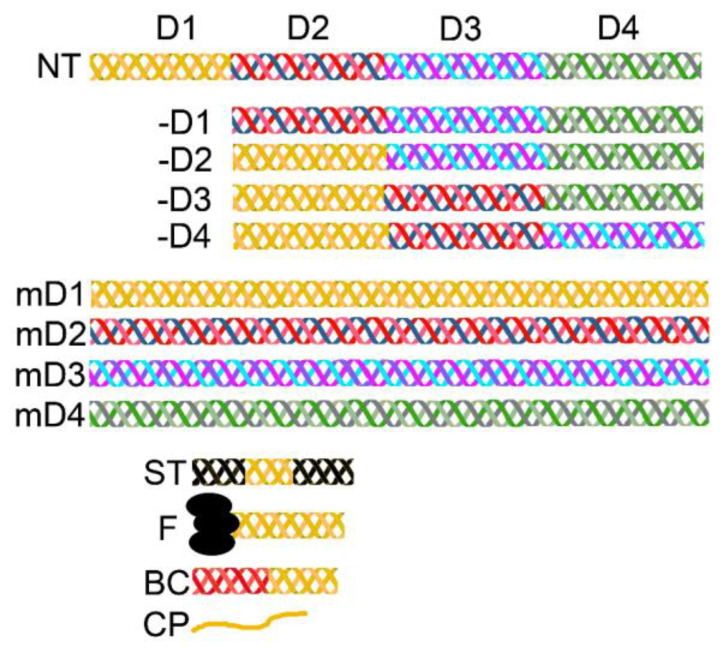
A schematic representation of recombinant collagen constructs studied by various research groups. NT; a native collagen molecule in which regions that correspond to the consecutive D-periods are indicated with different colors, -D1, -D2, -D3, and -D4; truncated collagen constructs in which specific D-periods were omitted, mD1, mD2, mD3, and mD4; collagen constructs comprising tandem repeats of specific D-periods, ST; a short collagen fragment stabilized by flanking it with stabilizing triple-helical peptides, F; a short collagen fragment stabilized by foldon domains, BC; a short collagen fragment stabilized by a fragment derived from bacterial collagen, CP; a recombinant or a synthetic linear peptide derived from collagen.

The successful expression of recombinant collagens opened an opportunity to produce not only native-like collagen constructs but also customized variants with modified sequences (Figure 4). Both groups of collagen constructs offer potentially attractive collagen-based materials for use in basic research and the commercial sector. The basic research area focuses on understanding the biological roles and the structure–function relation of various collagen types and their defined domains. The main interest of the commercial sector includes the production of bulk amounts of collagen materials for use in biomedicine and pharmaceutical, food, and cosmetic industries. Although the commercial sector focuses primarily on native-like recombinant collagens, in the future, variants with customized characteristics, including thermostability and the ability to interact with specific ligands, may offer significant advantages over their native-like counterparts.

Technologies to produce recombinant collagens for both research and commercial purposes have evolved significantly over the last three decades. Initially, the main focus was on engineering and expressing DNA constructs encoding the native-like human collagens, most notably collagen I. The initial challenges of expressing stable human collagen I molecules with the correct 2:1 ratio of the α1(I) and the α2(I) chains were solved so that human recombinant collagen types, both heterotrimeric and homotrimeric, can now be readily produced [73,93]. Similarly, Pihlajamaa et al. reported a successful expression of heterotrimeric collagen IX with the expected chain composition [97].

Studies on the mechanisms of binding interactions of collagens with cells demonstrated that triple-helical regions include distinct domains, usually formed by only a few amino acid residues, that interact specifically with collagen-specific cell receptors, including integrins, DDRs, GPVI, or LAIR-1 (Figure 2). These receptors not only recognize defined collagen sites, but some of them (e.g., GPVI) require collagen I and collagen III molecules in their fibrillar form. Although some of the collagen-specific receptors may also bind to the linear peptides (i.e., not triple-helical) corresponding to the native binding sites, the binding interactions are usually weak and may not trigger tissue-specific cell responses [98].

Because of those site-specific binding interactions that control many processes, including cell attachment, migration, and proliferation, scientists developed a concept of producing biologically active collagen fragments instead of the full-length native-like collagen molecules. The premise for this concept is that expressing or synthesizing short active collagen fragments would provide useful biomaterials with tissue-specific cell signaling properties [99,100,101,102,103,104,105,106,107,108,109].

The need for the triple-helical structure of the cell signaling domains and the fact that they are present in vivo in the context of fibrils, however, make the usefulness of the above concept uncertain. Although the scientific literature describes testing the utilities of many collagen-derived recombinant fragments to serve as biomaterials for tissue engineering and wound-healing applications, thus far, these constructs have provided useful experimental tools but have not been applied clinically.

In one example, however, a linear, i.e., non-triple helical, peptide, called P-15, is utilized as an ingredient in a bone graft material (i-FACTOR, CeraPedics Inc., Westminster, CO, USA) [110,111]. The P-15 peptide is derived from the collagen I site characterized by a relatively low content of the triple helix-stabilizing hydroxyproline residues [112]. Considering that this peptide does not include any known sites for binding cell receptors, mechanisms of its claimed biological activity remain unknown. Consequently, not knowing its mechanism of action, it is difficult to fully comprehend the benefits of using P-15-containing materials to promote bone tissue repair.

Similarly, a commercial product, referred to as recombinant collagen peptide (RCP, Cellnest; Fujifilm), that includes the Arg-Gly-Asp (RGD) peptide, has been used in various experiments to promote the regeneration of tissues, including skeletal, pancreatic, vascular, and others [69,113,114,115]. Although the RGD sequence is a part of the binding sites of many integrins, it is not considered the canonical binding site for the collagen-specific integrins α1β1 and α2β1. Moreover, the RGD sequence is not collagen specific. Indeed, it is present in many proteins, including fibronectin, vitronectin, fibrinogen, thrombospondin, entactin, and many other macromolecules. Therefore, due to the lack of a collagenous character, the RGD-based materials should not be classified as collagen mimics. Nevertheless, the RGD peptides must be presented in the form of organized clusters to show optimal cell-binding properties [116,117,118]. This need for organization of the RGD motifs makes the engineering of useful ECM architectures a challenging problem.

In addition to collagen-derived peptides with random conformations, researchers produce short collagen fragments with the triple-helical structure (Figure 4). Because short collagen triple helices are usually unstable and unfold at body temperature, scientists developed various techniques to stabilize them. In one example, they flanked collagen-derived sequences of interest with those corresponding to the triple helix-stabilizing Gly-Pro-Pro repeats [119,120,121]. Alternatively, hybridizing the peptides of interest with stabilizing sequences may maintain their triple-helical structures [122]. Moreover, scientists linked the collagen-derived sequences to the foldon domain’s fragments from bacteriophage T4 fibritin [123,124]. As the foldon region has a natural ability to form trimers, its presence stabilizes the triple helices formed by the assembly of short collagen peptides (Figure 4).

Furthermore, researchers have explored the possibility of producing relatively short collagen-like fragments fused with bacteria-derived triple-helical peptides. Unlike mammalian collagens, the bacterial collagen-like triple helices remain stable at high temperatures, despite the absence of hydroxyproline residues [55]. Scientists demonstrated that these bacterial collagens are safe and interact with some integrins [125]. Because of their stability, biocompatibility, and potential large-scale production, scientists believe that bacterial collagens hold promise for some biomedical applications (Figure 4) [91,126,127,128].

Although non-native sequences stabilize short collagen fragments, their presence may complicate the clinical approval of these constructs. One approach to circumvent this problem is to engineer the normal-length collagen-like constructs, comprising tandem repeats of selected native domains (Figure 4) [109]. Studies demonstrated that linking short domains into molecules whose length matches native collagens does not alter their ability to form triple-helical structures. However, some of these constructs have low thermostability, which renders them unusable in tissue engineering approaches [107]. Moreover, while some of these constructs retain the ability to aggregate into fibrillar structures, others cannot form proper fibrils [78]. Even though novel tandem-repeat variants have been used as research tools for mapping binding domains for the collagen-specific receptor, defining regions that drive fibrillogenesis, and as delivery vehicles for therapeutic cells, they have not yet been applied clinically [78,106,108,129].

Despite potential problems with the stability of some collagen constructs and the ability to form fibrils, they may still be used to form useful materials. For instance, introducing random chemical crosslinking stabilizes collagen constructs so that they can function at body temperature. Furthermore, to enable the formation of fibrillar structures, researchers use various techniques, including electrospinning and magnetic alignment of collagen molecules [130,131].

### 4.4. Proposed Biomedical Applications of Recombinant Collagen Constructs

Although this paper does not intend to provide exhaustive details for all published concepts on utilizing recombinant collagen constructs, it presents distinct categories of tested applications, including tissue repair and engineering, drug delivery, and protein replacement therapies (Table 2).

Recent literature indicates that scientists have tested the potential of recombinant collagens and their fragments as scaffolds and fillers in tissue engineering and repair approaches. It is worth noting that those tests aimed at defining the fundamental utilities of recombinant collagens in simple experimental models, with only a relatively few transitioning toward more relevant animal-based studies or clinical applications.

Researchers fabricated the scaffolds as porous sponges, fibrils, and membranes in 3D configurations to better support cell attachment and growth. Advances in the fabrication of scaffolds, electrospinning methods to align fibrils, and 3D printing technology have opened new possibilities to create organized scaffolds as well as tissue-like bioprinted constructs that include cells [113,130,132,133].

Because of the gelling properties of collagen constructs, they may be used to make injectable liquid materials that solidify at body temperature. This property allows the creation of scaffolds with proper shapes directly at the injury sites. Furthermore, the gels offer drug-delivery and cell-delivery vehicles that shield their cargo and release it in a controlled way. In one example, Confalonieri et al. employed a commercially available recombinant collagen peptide containing the RGD sequence as a material to form microspheres to support the growth of mesenchymal stromal cells [115]. Similarly, scientists proposed recombinant collagen-based hydrogels to regenerate damaged heart tissue [75].

Although recombinant collagens are not widely utilized for clinical, cosmetic, and pharmaceutical applications, and because they are used primarily in research applications performed in vitro, in cell culture conditions, and in animals, some companies started to introduce products fabricated from those proteins. In one notable example, the full-length collagen I expressed in tobacco plants is utilized in clinically applied products CE marked and approved for sale in Europe and Israel. These products (CollPlant Ltd.) include a flowable gel construct (VergenixFG) for wound dressing and a material for the treatment of tendinopathy (VergenixSTR) [95,96,134].

Moreover, recombinant gelatin was once considered promising for use in pharmaceutical formulations, including vaccines and drug-delivery capsules, but it has not yet found its way into the market. According to the summary list of vaccines licensed for use in the USA, hydrolyzed gelatin of the porcine origin serves as the stabilizer in most vaccine formulations [135].

## 5. Recombinant Collagens for Protein Replacement Therapies

Recombinant collagens could potentially be used in a protein replacement therapy in patients who suffer from genetic disorders due to mutations in collagen genes (Table 3). Although these disorders are very heterogeneous with no clear genotype–phenotype relationship, most are characterized by a decrease in a collagen type affected by a mutation [17]. Thus far, tests of the utility of the collagen replacement therapies have focused on diseases that affect basement membranes of the skin and kidney. In the first case, researchers targeted collagen VII, and in the second case, they targeted collagen IV [157,158,159,160].

While most collagen mutations are single amino acid substitutions that allow mutant collagen chain production, some mutations create premature stop codons that entirely prevent biosynthesis. For instance, in recessive dystrophic epidermolysis bullosa (RDEB), a form of blistering skin disease, collagen VII may be absent. The lack of collagen VII in the basement membranes, where this protein plays a pivotal mechanical role, causes severe skin fragility and blistering of the esophagus and the eye surface. Furthermore, the progressive nature of RDEB leads to excessive skin damage, scarring, contractures, and fusion of the fingers, and RDEB patients may also develop squamous cell carcinoma [161].

Despite some experimental approaches to treat RDEB, there are no therapies to cure this disorder. To date, researchers have tested the following methods to introduce normal collagen VII into diseased tissues: (i) protein replacement therapies by delivery of the *COL7A1* gene that encodes normal collagen VII chains, and (ii) direct delivery of recombinant collagen VII protein [162].

Considering the direct delivery of collagen VII, scientists produced the recombinant form of this protein and then injected it directly into collagen VII-null mice. When Remington et al. injected recombinant collagen VII into the skin of collagen VII-deficient mice, they observed site-specific accumulation of this protein in the area of the dermal basement membrane zone and the formation of collagen VII assemblies, namely the anchoring fibrils [158]. Despite injecting collagen VII into the mice lacking this protein, the authors did not observe the formation of anti-collagen VII antibodies. In another study, recombinant collagen VII was injected into the bloodstream of the collagen VII-null mice and observed that, as in the case of the intradermal injection, the exogenous recombinant collagen VII accumulated in proper tissue locations, including the dermal–epidermal junction, tongue, and esophagus [157].

Despite these promising preliminary results in mice, the direct delivery of recombinant collagen VII has not moved from bench to bed, such as to improve the structural integrity of tissues among patients with RDEB. The reasons for this lack of progress in protein replacement therapy for RDEB are not clear. However, it seems that the direct delivery of collagen VII into cavities of connective tissues is challenging. One of the potential problems might be a large Stokes radius of collagen VII, one of the largest known proteins in the human body, present in solution. Thus, it is unlikely that collagen VII, or other collagen types, could readily diffuse to the target tissue sites. This problem is amplified by the fact that a high-affinity collagen VII–collagen VII binding interaction promotes unwanted aggregation [84].

Furthermore, before self-assembly into functional anchoring fibrils, the procollagen C proteinase must cleave collagen VII [84]. In brief, procollagen C proteinase has to cleave a portion of the C-terminal propeptide before processed molecules can form anti-parallel dimers stabilized by the disulfide bonds. Subsequently, the dimers must bundle together and arrange into the anchoring fibrils that interlace with the dermal and epidermal matrices. It is unlikely that these intricate processes occur efficiently with exogenous collagen VII delivered by transdermal or intravenous injections. Recent research by Supp et al. supports this notion: utilizing relevant models, the authors demonstrated that for the anchoring fibrils to form and function correctly within the dermal–epidermal junction, both epidermal keratinocytes and dermal fibroblasts must produce collagen VII [163].

An additional potential limitation of the direct delivery of collagen VII is its half-life. As demonstrated by Khül et al., the collagen VII half-life is about one month, which means this protein would have to be injected frequently in large quantities to have any meaningful positive, long-term effects [164].

Furthermore, it is unclear what effect the intravenous injection of collagen VII has on platelet aggregation. Although experiments in vivo demonstrated that this collagen type does not aggregate platelets as strongly as fibril-forming collagens, it can still activate them. Thus, we cannot exclude a possibility that collagen VII in the bloodstream will not trigger clot formation [165].

Due to the concerns presented above for direct collagen VII delivery, clinical application of protein replacement therapies for other collagen types may be subject to similar problems. For instance, scientists have considered collagen IV delivery, via local or systemic routes, to treat Alport syndrome caused by genetic aberrations of collagen IV [159]. However, scientists have not yet determined the efficacy of this approach thus far, and concerns persist regarding aggregation, diffusion, and potential activation of platelets.

Choosing the potential treatment time is an additional consideration for using replacement therapies to treat disorders caused by a mutation in collagen genes. As collagens are needed to form templates of tissues from early embryonic development, it is unclear whether the post-natal delivery of a therapeutic recombinant collagen type would restore and maintain functional target tissue. As demonstrated in a mouse model of spondyloepiphyseal dysplasia caused by a mutation in collagen II, only early embryonic interventions led to normal skeletal tissues. In contrast, late embryonic and post-natal interventions did not improve these tissues in any significant way [166,167].

Ultimately, clinical trials are needed, such as those planned by Phoenix Tissue Repair, Inc. to determine the safety and efficacy of protein replacement therapy to treat RDEB patients harboring collagen VII mutations [168]. If successful, these trials may open a possibility for using replacement therapy for diseases caused by mutations in other collagen types. Table 3 highlights experimental replacement therapies that utilize recombinant collagens.

## 6. Constraints on Implementing Recombinant Collagen Technology in Clinical Applications

Despite developing technologies to design and produce recombinant collagens with native structures and collagen-derived constructs, these proteins have not so far succeeded in the clinical marketplace. Although it is beyond the scope of this review to analyze the specific reason for this situation, possible causes include the following points:

- There is no clear consensus on a system for large-scale recombinant collagen production that would be accepted by the regulatory agencies responsible for approving biologics for commercial clinical use. The production systems encompass different organisms, including bacteria, mammalian cells, insect cells, yeast, transgenic animals, and transgenic plants.
- There is no consensus on the most relevant form of recombinant collagens needed in the market. While some studies consider production and application of the native-like collagens, others propose to manufacture and use collagen-derived synthetic linear peptides, triple-helical fragments, and genetically engineered collagen-inspired constructs. For instance, it is not clear whether recombinant gelatin will be manufactured from the full-length recombinant collagens or selected recombinant fragments.
- Because of the wide span of potential biomedical applications of recombinant collagens, ranging from drug delivery, tissue engineering, wound healing, and protein replacement therapies, there is no identifiable leading product that could attract the attention of the market. Many different collagen types are needed for medical applications in distinct tissues and organs, amplifying this problem.
- Although some concerns about the safety of animal-derived collagen materials exist, pharmaceutical, cosmetic, and food industries continue to use them. Furthermore, because these materials are readily available from tissues of isolated animal herds, they are likely less expensive than recombinant collagens whose production requires advanced technologies.
- Even with a few companies’ early interest in producing and delivering large amounts of recombinant collagens and gelatins, no commercial products are widely available on the market. This situation may indicate that the market’s needs differed from companies’ expectations about recombinant collagens’ commercial potential.
- Tissue engineering is a crucial proposed use for recombinant collagen variants. Despite the promising preclinical results of many tissue-engineered medical products, only a few have had success in the clinic thus far [169]. Consequently, it is likely that the primary potential beneficiary of recombinant collagen technology, i.e., the tissue engineering industry, does not clamor for novel recombinant collagen-based materials in any significant way.

Considering these factors, it is likely that technologies to produce and implement recombinant collagen-based products face an uphill battle in finding a permanent place in the market. Meanwhile, animal-derived collagens will most likely continue to provide the bulk of material needed for biomedical, pharmaceutical, food, and cosmetic industries. Recombinant collagens, however, will expand their role as a valuable research tool needed to study not only the family of collagenous proteins but also on the extracellular matrix as a whole.

## Figures and Tables

**Table 1:** A summary of the expression systems for production of recombinant collagens.

Expression System	Examples of Collagen Constructs	Requirement for Co-Expression of P4H (N = No, Y = Yes)	Industrial-Scale Production (N = No, Y = Yes)	Commercial Evaluation (N = No, Y = Yes)	References
Mammalian cells (HT1080, CHO, HEK293, NIH3T3)	Native-like human procollagens, including procollagen I, procollagen II, collagen VI, procollagen VII. Fragments of procollagens, including mini-collagen II, mini-collagen I homotrimer, mini-collagen VII, C-terminal propeptides of procollagen III, and fragments of collagen IV	N	N	N	[4,50,53,83,84,85,86,87,88]
Insect cells	Native-like collagens including collagen I, collagen II, collagen III, collagen IX, collagen	N	N	N	[89,90]
Mammary glands of transgenic mice	Collagen I homotrimer	N	N	N	[62]
*Escherichia coli*	Human-derived mini-collagen III, collagen fragments, including C propeptide of collagen XVIII, and fragments of collagen I	Y	N	N	[61]
*Escherichia coli*	Collagen fragments stabilized by bacterial collagen-like sequences	N	N	N	[91,92]
Yeast cells	Native-like human collagen I, collagen III, gelatin	Y	Y	Y	[28,60,72,93]
Transgenic plants	Native-like human collagen I	Y	Y	Y	[59,77,94,95,96]

**Table 2:** Examples of recombinant collagen-based constructs and their potential applications in tissue repair and engineering.

Collagen Construct	Expression System	Proposed Application	Experimental Tests	Applied Products (N = No, Y = Yes)	References
Full-length native-like collagen II, collagen VII	Mammalian cells HT1080, CHO	Cartilage engineering, protein replacement in patients harboring mutations in collagen VII, research tool	In vitro, mouse	N	[88,105]
Truncated and modified collagen II variants, truncated collagen VII	Mammalian cells HT1080, HEK293	Cartilage engineering, research tool	In vitro, mouse	N	[16,83,109]
Full-length native-like human collagen I, collagen III	Yeasts	Fabrication of scaffolds and hydrogels to repair damaged tissues	Mouse	N	[75]
		Hemostatic materials	Rabbit	N	[136]
		Implants to regenerate cornea	Human	N	[137,138]
Modified collagen III	Yeasts	Materials with increased thermostability	In vitro	N	[139]
		Materials with customized collagen III sequences for support of stem cells	In vitro	N	[129]
Collagen III constructs containing integrin-binding sites from collagen I and laminin	Yeasts	Scaffolds to support neural progenitor cells	In vitro	N	[129]
Non-triple helical collagen I fragment	Yeasts	Scaffolds for tissue regeneration	In vitro	N	[99]
		Scaffolds for transplantation of pancreatic islets	Mouse	N	[140]
		Grafting material for bone regeneration	In vitro	N	[115,141,142,143,144]
Native-like collagen II	Yeasts	Hydrogel to support chondrogenesis of mesenchymal stromal cells	In vitro	N	[145,146]
Collagen I fragment fused with (Pro-Gly-Pro)_9_ peptides	Yeasts	Gelatin mimetic	In vitro	N	[31]
Full-length human collagen III	Bacteria	NA	In vitro	N	[70]
Collagen III fragments fused with bacteria-derived collagen-like proteins	Bacteria	Inhibitors of DDR signaling	In vitro	N	[147]
Tandem repeats of the (GAPGAPGSQGAPGLQ) fragment	Bacteria	Material to deliver BMP-2 for bone repair	Mouse	N	[148]
Tandem repeats of (GPP) fragment		Fabrication of biocompatible surfaces	In vitro	N	[149]
Fragment of turtle-derived collagen	Bacteria	Antioxidant material	In vitro	N	[150]
Collagen III-derived fragments	Bacteria	Treatment of vaginal atrophy	Rat	N	[151]
Full-length native-like human collagen I	Tobacco	Wound dressing materials		Y	[134]
		Matrices for ovarian grafting	Mouse	N	[152]
		Scaffolds for bone and skin repair	In vitro	N	[153,154]
		Electro-spun fibrils for tendon-repair materials	In vitro	N	[155]
		Injectable material combined with platelet-rich plasma for treatment of lateral epicondylar tendinopathy	Human	Y	[76]
Short collagen-derived linear peptides	Bacteria, yeast (recombinant technology) and chemical synthesis	Osteogenic material	In vitro, human	Y	[68,69,110,115,156]

**Table 3:** Experimental protein replacement therapies with the use of recombinant collagens.

Disease	Collagen Target	Experimental Model	Clinical Tests	Applied Clinically	References
Dystrophic epidermolysis bullosa	Collagen VII	Intradermal or intravenous delivery of recombinant collagen VII into mice	Y	N	[168]
Alport syndrome	Collagen IV	Systemic delivery	N	N	[159,160]
